# A New Norsesquiterpenoid from a Bornean Soft Coral Genus *Nephthea*

**DOI:** 10.3390/molecules14114591

**Published:** 2009-11-10

**Authors:** Takahiro Ishii, Hiroshi Matsuura, Zhan Zhaoqi, Charles Santhanaraju Vairappan

**Affiliations:** 1 Laboratory of Natural Products Chemistry, Institute for Tropical Biology and Conservation, Universiti Malaysia Sabah, 88999 Kota Kinabalu, Sabah, Malaysia; E-Mail: ishii_t@ums.edu.my (T.I.); 2 Graduate School of Environmental Science, Hokkaido University, Sapporo 060-0810, Japan; E-Mail: matsuura@ees.hokudai.ac.jp (H.M); 3 Shimadzu (Asia Pacific) Pte Ltd, 16 Science Park Drive, #01-01, The Pasteur Singapore Science Park, 118227 Singapore; E-Mail: zhaoqi@shimadzu.com.sg (Z.Z.)

**Keywords:** germacrane-type sesquiterpenoid, *Nephthea* sp., Nephtheidae, soft coral

## Abstract

A new germacrane-type norsesquiterpenoid, 1-acetoxy-germacra-5*E*,10(14)-diene-4-one (**1**), as well as three known compounds, were isolated from the organic extracts of a Bornean soft coral *Nephthea* sp. Their structures were elucidated on the basis of spectroscopic data analysis.

## Introduction

**Figure 1:**
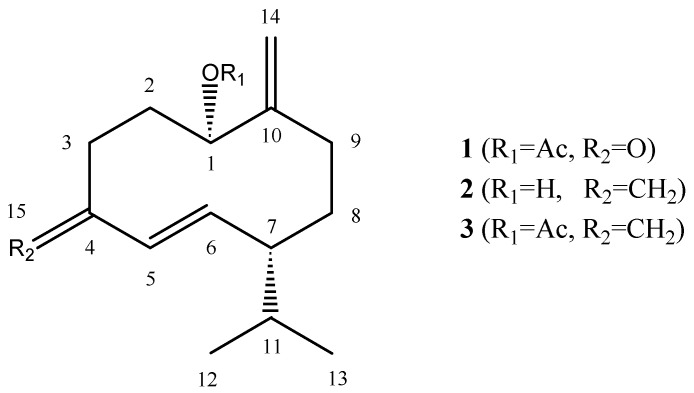
Structures of compounds **1**-**3**.

Soft corals of the genus *Nephthea* (Alcyonacea, Nephtheidae) contain a variety of bioactive metabolites such as sesquiterpenes, diterpenes and steroids [1,2,3,4,5,6,7,8,9,10,11,12,13], and they are widely distributed in the coastal waters of Sabah, Malaysia. However, there have been few reports on chemical investigation of Malaysian soft corals to date. To our knowledge, our previous chemical investigation on the soft coral species belonging to genus *Nephthea* constituted the first report of isolation and identification of secondary metabolites from Malaysian soft corals [14]. Prompted by our interest in discovering novel compounds from this genus, we investigated a specimen collected from Sibuan Island, Sabah. The methanol extract gave a new germacrane-type norsesquiterpenoid, 1-acetoxy-germacra-5*E*,10(14)-diene-4-one (**1**), along with three known compounds: germacra-4(15),5*E*,10(14)-trien-1-ol (**2**) [9,15,16], 1-acetoxy-germacra-4(15),5*E*,10(14)-triene (**3**) [9,15,16] and 24-methylenecholesterol (**4**) [17] (Figure 1). In this paper we report the isolation and structural determination by spectroscopic methods of the new norsesquiterpenoid.

## Results and Discussion

Compound **1** was isolated as a colorless oil. HR-MS gave a molecular formula of C_16_H_24_O_3_ with five degrees of unsaturation. The ^1^H- and ^13^C-NMR spectral data (Table 1) indicated the presence of an acetoxy group [δ_C_ 170.2 (s), 21.3 (q); δ_H_ 1.98 (3H, s)], an α,β-conjugated keto group [δ_C_ 203.0 (s), 154.0, 130.1; 6.40 (1H, dd, *J* = 15.8, 10.6 Hz), 6.01 (1H, d, *J* = 15.8 Hz)], an isopropyl residue [δ_C_ 31.6 (d), 20.9 (q), 20.6 (q); δ_H _1.59 (1H, dq, *J* = 13.7, 6.8 Hz), 0.84 (3H, d, *J* = 6.8 Hz), 0.93 (3H, d, *J* = 6.8 Hz)], an oxymethine [δ_C_ 76.2 (d); δ_H _4.99 (1H, dd, *J* = 11.6, 4.1 Hz)], and a terminal methylene moiety [δ_C_ 115.3 (t); δ_H _5.35 (1H, d, *J* = 2.0 Hz), 5.20 (1H, d, *J* = 2.0 Hz)]. According to the molecular formula and the functionalities mentioned above, compound **1** was suggested to be a monocyclic norsesquiterpene.

**Figure 2:**
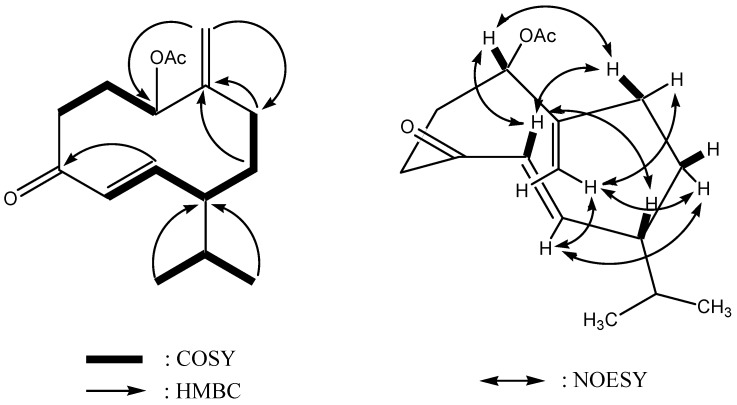
Selected 2D NMR correlations for **1**.

Assignments were carried out based on ^1^H–^1^H COSY, HSQC and HMBC spectra data. ^1^H–^1^H COSY experiment revealed the sequences of the correlations depicted by the bold lines in Figure 2. HMBC correlations from H-14 to C-1 and C-9 were important to confirm that the exomethylene moiety can be inserted between C-1 and C-9. HMBC correlations from H-12 and H-13 to C-7 indicated the isopropyl group was attached directly to the methine (C-7). In addition, correlations from H-6 to C-4 allowed us to confirm the α,β-conjugated keto group. The chemical shift for C-1 (δ_c_ 76.2; δ_H _4.99) indicated that the acetoxy group was attached to the oxymethine carbon at C-1. The ketone moiety (C-4) was suggested to be adjacent to C-3 (δ_C_ 36.2). Based on these findings, the gross structure of **1** was determined as shown in Figure 1.

**Table 1:** ^1^H-NMR and ^13^C-NMR spectral data of compound **1** (recorded at 600/150 MHz in CDCl_3_; δ in ppm, *J* in Hz).

Position	^13^C	^1^H (*J* in Hz)
1	76.2 (CH)	4.99 (dd, *J* =11.6, 4.1 Hz, 1H)
2	28.6 (CH_2_)	2.13 (m, 1H) (Ha)
		1.99 (m, 1H) (Hb)
3	36.2 (CH_2_)	2.98 (ddd, *J* =12.4, 12.4, 4.8 Hz, 1H) (Ha)
		2.26 (ddd, *J* =12.4, 4.8, 4.8 Hz, 1H) (Hb)
4	203.0 (C)	
5	130.1 (CH)	6.01 (d, *J* =15.8 Hz, 1H)
6	154.0 (CH)	6.40 (dd, *J* =15.8, 10.6 Hz, 1H)
7	53.2 (CH)	1.96 (m, 1H)
8	34.4 (CH_2_)	2.09 (m, 1H) (Ha)
		1.67 (m, 1H) (Hb)
9	33.5 (CH_2_)	2.51 (ddd, *J* =15.1, 6.8, 1.4 Hz, 1H) (Ha)
		1.74 (m, 1H) (Hb)
10	147.9 (C)	
11	31.6 (CH)	1.59 (dq, *J* =13.7, 6.8 Hz, 1H)
12	20.9 (CH_3_)	0.84 (d, *J* =6.8 Hz, 3H)
13	20.6 (CH_3_)	0.93 (d, *J* =6.8 Hz, 3H)
14	115.3 (CH_2_)	5.35 (d, *J* =2.0 Hz, 1H) (Ha)
		5.20 (d, *J* =2.0 Hz, 1H) (Hb)
OAc	170.2 (C)	
	21.3 (CH_3_)	1.98 (s, 3H)

The relative stereochemistry of **1** was determined by the coupling constants in the ^1^H-NMR spectrum as well as the NOESY experiment. The *E*-configuration for the double bond between C-5 and C-6 was determined by the high value (*J* = 15.8 Hz) of the coupling constant between the pertinent olefinic protons. Furthermore, as shown in Figure 2, the NOESY correlations observed between H-1/H-5, H-1/Hb-9, Ha-3/H-6, Ha-3/Ha-14, H-5/H-7, H-5/Hb-9, H-6/Hb-8, H-6/Ha-14, H-6/Hb-14, Hb-8/Hb-14, Ha-9/Hb-14 revealed H-1 and H-7 were located on the same orientation of the molecule. Unfortunately, the absolute stereochemistry of **1** could not be determined from the nature of its optical rotation (**1**: [α]^23^_D_ –16.0, **2**: +6.6, **3**: +78.2) or based on biogenesis consideration since compound **2 **has been reported to exist in Nature as a mixture of enantiomers in various ratios [15]. Therefore, compound **1** was identified as 1-acetoxy-germacra-5*E*,10(14)-diene-4-one. All compounds were evaluated for antimicrobial activity against 10 human pathogenic bacteria. Compound **4** showed weak activity against *Proteus mirabilis*, *Salmonella enteridis* and *S. thyphymunium* at 30 μg/disc, however, compounds **1**-**3** were inactive at 30 μg/disc.

## Conclusions

As a part of our chemical investigation on Malaysian soft corals, a new germacrane-type norsesquiterpene, 1-acetoxy-germacra-5*E*,10(14)-diene-4-one (**1**), was isolated from *Nephthea* sp. collected from Sibuan Island, Sabah, along with three known compounds. Their structures were established on the basis of spectroscopic evidence. To our knowledge, the two known compounds **2** and **3** were isolated from the genus *Nephthea* for the first time. These results will lead us to find further novel secondary metabolites in Malaysian soft corals.

## Experimental Section

### General

Optical rotations were measured on an AUTOPOL IV automatic polarimeter (Rudolph Research Analytical). ^1^H-NMR (600 MHz) and ^13^C-NMR (150 MHz) spectra were recorded with a JEOL ECA 600, with TMS as internal standard. HR-ESI-TOFMS spectrum was obtained with LCMS-IT-TOF (Shimadzu). HPLC was conducted on a Waters 600 using UV detector and Luna 5μ C18(2) 100A (10.0 × 250 mm). Preparative TLC was performed with silica gel plate (Merck, Kieselgel 60 F_254_). Silica gel (Merck, Kieselgel 60, 70-230 mesh) was used for column chromatography. Analytical TLC was performed on Merck Kieselgel 60 F_254_. Spots were visualized by UV light or by spraying with a 5% phosphomolybdic acid-ethanol solution.

### Biological material

Specimen of *Nephthea* sp. was collected from Sibuan Island, Sabah (4^o^39’089’’N, 118^o^39’579’’E), on March 8, 2008. The gross morphological features of this soft coral were very similar to those of *Nephthea erecta*. The voucher specimen (MAR37768BOR) was deposited in the BORNEENSIS Collection of Institute for Tropical Biology and Conservation, Universiti Malaysia Sabah.

### Extraction and isolation

The fresh soft coral (324 g wet wt) was extracted with MeOH (1 L) at room temperature for 7 days. The crude extract was evaporated under reduced pressure and the residue (1.46 g) was partitioned between EtOAc and H_2_O (each 500 ml). The EtOAc fraction was further partitioned with hexane and 90% MeOH. The hexane fraction (900 mg) was chromatographed on a Si gel column using hexane and EtOAc system of increasing polarity as eluant to yield six fractions (Fr. 1-6). Fraction 2 (101 mg) eluted with hexane/EtOAc (9:1) was further subjected to reversed-phase HPLC (Luna 5μ C18(2) 100A) with 70% MeCN to give compound **1** (1.0 mg). In addition, fraction 2 was submitted to preparative TLC with toluene to yield compound **3** (6.5 mg). Fraction 5 (120 mg) was further separated by repeated preparative TLC with CHCl_3_ and hexane/EtOAc (3:1) to give compounds **2** (5.5 mg) and **4** (28.9 mg).

### Characterization of 1-acetoxy-germacra-5E,10(14)-diene-4-one (**1**)

Colorless oil; [α]^23^_D_ –16.0 (*c* 0.05, CHCl_3_); HR-TOFMS *m*/*z* 265.1809 [M+H]^+^ (calcd. for C_16_H_25_O_3_, 265.1798); ^1^H-NMR and ^13^C-NMR spectral data: see Table 1.

### Antibacterial bioassay

The antimicrobial bioassays for the isolated compounds were carried out using 10 strains of human pathogenic bacteria: *Escherichia coli* (CSV01-08), *Listeria monocytogenes* (CSV02-08), *Proteus mirabilis* (CSV03-08), *Pseudomonas aurelis* (CSV04-08), *Salmonella* sp*.* (CSV05-08), *Salmonella typii* (CSV06-08), *Salmonella enteridis* (CSV07-08), *Salmonella thyphymunium* (CSV08-08), *Staphylococcus aereus* (CSV09-08) and *Vibrio cholerae* (CSV10-08). The assay was performed as previously described [18].
